# Impact of Storage Lesion on Post-transfusion Rise in Hemoglobin

**DOI:** 10.7759/cureus.2952

**Published:** 2018-07-09

**Authors:** Muhammad Sardar, Nasreen Shaikh, Jack Ansell, Aasems Jacob, Srujana Yada, David B Kelly, Mohankumar Doraiswamy, Wahab J Khan, Faiz Anwer, Margaret HH Eng

**Affiliations:** 1 Internal Medicine, Monmouth Medical Center, Long Branch, USA; 2 Hematology, Monmouth Medical Center, Long Branch, USA; 3 Pediatrics, Newark Beth Israel, Newark, USA; 4 Hematology and Oncology, University of Arizona, Tucson, USA

**Keywords:** storage lesion, transfusion, rise in hemoglobin

## Abstract

Background: The storage lesion is defined as the set of changes that occur in red blood cells (RBCs) during storage. Studies have shown that a prolonged storage period of RBCs is associated with increased destruction after transfusion. The aim of this study is to determine the impact of the storage lesion on the efficacy of RBC transfusions by comparing the mean rise in the hemoglobin of patients who received new vs old blood.

Methods: We did a retrospective chart review of all patients who received a single unit of pure red blood cell (PRBC) transfusion in a three-month period. Patients with hemolytic anemia and active bleeding were excluded. The storage lesion was estimated by calculating the number of days to expiration on the day of transfusion. Median days to expiration was calculated to be 11 days. Patients were divided into two groups based on days to expiration. Group A included patients who received old blood (days to expiration: 0-11) and group B included patients who received new blood (days to expiration: 11-38). The mean rise in hemoglobin between the two groups was compared using the paired t-test.

Results: The baseline characteristics of both groups were similar. There was no statistically significant difference in the mean rise in hemoglobin (1.01 vs 1.08- p-value 0.298), hematocrit (3.37 vs 3.61- p-value 0.249), and RBC count (0.42 vs 0.44- p-value 0.097) in the group that received old blood vs new blood, respectively.

Conclusion: An RBC transfusion with a shorter storage period does not increase hemoglobin more than RBC with a longer storage period.

## Introduction

The storage lesion refers to the set of biochemical and structural changes that occur during the storage of red blood cells (RBCs) [1]. The transfusion of RBCs after a prolonged storage period leads to increased RBC lysis, exaggerated inflammatory response, and nitric oxide (NO) scavenging from free hemoglobin and microparticles [2]. The deleterious effects of the storage lesion at a molecular level are well-established but the potential clinical relevance is unclear. Earlier studies reported increased mortality associated with transfusing older blood, but recent trials have reported no such effect of the storage lesion in critically ill patients [3-4]. Multiple trials have looked at the effect of the storage lesion on mortality and morbidity but little is known about its impact on the RBCs ability to raise post-transfusion hemoglobin. Blood products that have accumulated the storage lesion are more prone to hemolysis after transfusion and, hence, may impact the post-transfusion rise in hemoglobin. We hypothesize that if a relatively new pure red blood cell (PRBC) unit can achieve a higher rise in hemoglobin after transfusion, we can selectively transfuse new blood to patients requiring multiple transfusions and potentially limit the total number of transfusions required to reach a target hemoglobin.

## Materials and methods

We used the blood bank order report to identify 723 consecutive patients who received PRBC transfusions over a three-month period (from October 2017 to December 2017) at a community teaching hospital. We excluded patients who received more than one unit in a 24-hour period, patients with active overt bleeding within 48 hours of blood transfusion, medical history, and/or laboratory evidence of hemolytic anemia, patients who had a major transfusion reaction, or patients who received an intravenous fluid bolus on the day of transfusion, the latter to negate its dilutional effect. Patients who did not have hemoglobin and hematocrit checked before and after the transfusion within a 24-hour period were also excluded. A thorough retrospective chart review of all PRBC transfusion orders was done by five internal medicine residents and 198 orders (patients) were included in the final analysis. The storage lesion was estimated by calculating the number of days to expiration each PRBC unit had on the day of transfusion.

The median number of days to expiration on the day of transfusion was 11 days. We divided the patients into two groups based on the number of days to expiration of the PRBC unit each patient received. Patients who received blood close to its expiration date and, hence, relatively old blood (days to expiration from 0 to 11) were included in group A (n=99). Patients who received blood that was relatively new (days to expiration from 11 to 38) were included in group B (n=99). Baseline characteristics, including age, gender, height, weight, relevant blood count indices, and medical history, were compared. We calculated the mean pre-transfusion and the mean post-transfusion hemoglobin, hematocrit, and red blood cell count of all patients. To determine the effect of the storage lesion on efficacy, we compared the mean rise in hemoglobin, hematocrit, and RBC count between the two groups using the one-tailed t-test. All data were analyzed using SPSS 25.0 (SPSS Inc., Chicago, Illinois, US).

## Results

The baseline characteristics of patients in both groups were similar (Table 1).

**Table 1:** Patient characteristics SD - standard deviation; RBC - red blood cell count

Patient characteristics	Old blood (n=99)	New blood (n=99)	p-value
Age - mean (SD)	65.59 ± 18.8	65.46 ± 16.4	0.961
Male gender (n)	35	36	0.885
Height - mean (SD)	161.64 ± 19.52	165.95 ± 12.7	0.067
Weight - mean (SD)	72.53 ± 26.9	76.43 ± 19.7	0.246
Pre-transfusion hemoglobin -mean (SD)	7.41 ± 0.85	7.39 ± 0.74	0.844
Pre-transfusion hematocrit - mean (SD)	23.18 ± 2.79	23.09 ± 2.66	0.833
Pre-transfusion RBC count - mean (SD)	2.62 ± 0.44	2.67 ± 0.52	0.424
Post-transfusion hemoglobin - mean (SD)	8.39 ± 0.91	8.37 ± 0.85	0.846
Post transfusion hematocrit - mean (SD)	26.54 ± 2.96	26.34 ± 2.93	1.000
Post transfusion RBC count - mean (SD)	3.24 ± 0.41	3.09 ± 0.54	0.571
Hemoglobin recheck time after transfusion-mean hours (range)	9 (3-17)	8.47 (4-23)	0.387
Mean corpuscular volume (MCV)	90.01 ± 9.2	88.11 ± 10.26	0.177
Red blood cell distribution width (RDW)	18.36 ± 4.05	19.82 ± 5.32	0.034
Mean corpuscular hemoglobin concentration (MCHC)	31.71 ± 2.7	31.5 ± 1.8	0.556
Coronary artery disease n (%)	22 (22.2)	18 (18.2)	0.479
Chronic kidney disease n (%)	23 (23.2)	16 (16.2)	0.211
Cancer n (%)	36 (36.4)	48 (48.5)	0.084
Bone marrow suppression n (%)	7 (7.1)	11 (11.1)	0.322
Chronic inflammatory state n (%)	9 (9.1)	7 (7.1)	0.602
Infection n (%)	5 (5.1)	6 (6.1)	0.756
Number of transfusions in the last six months - mean (SD)	2.06 ± 3.95	3.33 ± 4.5	0.058

The mean pre-transfusion hemoglobin in the group that received old blood was 7.41 ± 0.85 gm/dl as compared to 7.39 ± 0.74 gm/dl with no statistically significant difference (p-value 0.844). Similarly, there was no significant difference in the mean pre-transfusion hematocrit (23.18 ± 2.79 % vs 23.09 ± 2.66 % - p-value 0.833) and RBC count (2.62 ± 0.44 X10^6^/µl vs 2.67 ± 0.52 X10^6^/µl - p-value 0.833) between the two groups. The mean post-transplant hemoglobin (8.39 ± 0.91 gm/dl vs 8.37 ± 0.85 gm/dl - p-value 0.85), hematocrit (26.54 ± 2.96 % vs 26.34 ± 2.93 % - p-value 1), and RBC count (3.24 ± 0.41 X10^6^/µl vs 3.09 ± 0.54 X10^6^/µl - p-value 0.571) were comparable in group A vs B, respectively. The median days to expiration of the PRBC unit was four in group A as compared to 16 in group B. There was no statistically significant difference in the mean time lapse between transfusion and rechecking hemoglobin between the two groups, i.e., nine hours (range: 3-17) in group A vs 8.47 hours (range:4-23) in group B. Patients who received new blood had a higher mean red blood cell distribution width (RDW) at the time of transfusion as compared to the group that received old blood (19.82 ± 5.32 % vs 18.36 ± 4.05 % - p-value 0.034). There was no difference in the prevalence of coronary artery disease (22 vs 18 - p-value 0.479), chronic kidney disease (23 vs 16 - p-value 0.211), cancer (36 vs 48 - p-value 0.084), infections (5 vs 6 - p-value 0.756) in the group that received old blood vs. new blood, respectively. Group A received a mean of 2.06 ± 3.95 blood transfusions in the previous six months as compared to 3.33 ± 4.5 in group B with no statistically significant difference.

The mean rise in hemoglobin after transfusing old blood was 1.01 gm/dl with no statistically significant difference from the mean rise in hemoglobin of patients who received new blood, i.e., 1.08 gm/dl - p-value 0.298. The mean rise in hematocrit and RBC count were also similar in both groups (3.37 % vs 3.61 % - p-value 0.249) and (0.42 X10^6^/µl vs 0.44 X10^6^/µl - p-value 0.097), respectively (Table 2).

**Table 2:** Post-transfusion rise in hemoglobin, hematocrit, and red blood cell count

Variable	Old blood	New blood	p-value
Mean rise in hemoglobin	1.01	1.08	0.2984
Mean rise in hematocrit	3.37	3.61	0.2498
Mean rise in red blood cell count	0.42	0.44	0.0979

## Discussion

To the best of our knowledge, this study is the first attempt to evaluate the effectiveness of the storage lesion on the post-transfusion rise of hemoglobin. Our study shows that patients who were transfused PRBCs with a shorter storage period did not have a higher rise in their hemoglobin, hematocrit, or RBC count within 24 hours post-transfusion compared to patients who received blood with a prolonged storage period. The storage lesion affects the post-transfusion viability of RBCs through various mechanisms. As the blood products are stored, the intracellular glucose level drops, resulting in the reduced production of adenosine triphosphate (ATP) and other important chemical mediators such as 2-3 diphosphoglycerate (2-3 DPG). These biochemical changes in the RBCs lead to an increased susceptibility to oxidative stress compromising membrane integrity and early hemolysis [5-6]. Moreover, the stored RBCs also undergo early exposure and the activation of removal signals present on the cell membranes that are identical to the physiological aging antigens. The activation of the removal signals leads to early immune recognition and the removal of red blood cells after transfusion [7-8].

Laboratory studies have shown evidence of increased in vivo hemolysis of RBCs that are transfused after an increased storage time. In a study by Luten et al., post-transfusion recovery (PTR), as measured by flow cytometry, was significantly higher when PRBC units with a shorter storage period (mean 5+/-2) were transfused as compared to units with a longer storage period (30 +/-3) in the same patient. The difference was attributed to the damage already incurred during the storage period, as most of the removal of RBC was during the first hour [9]. In a separate study by Hod et al., there was a significantly higher rise in total bilirubin in patients who received older blood whereas there was no difference in the other parameters of hemolysis (i.e., lactate dehydrogenase and haptoglobin) [10]. The biochemical changes during the storage period appear to be more pronounced than the normal aging of red blood cells in vivo, as evident by the increased hemolysis in the immediate post-transfusion period. Studies have shown that between 5% and 10% of RBCs disappear in the first 24 hours after transfusion followed by a linear disappearance curve [11]. The average lifespan of RBCs that are stored for 42-45 days is around 90 days as compared to 120 days in normal RBCs produced by the individual’s own hematopoietic stem cells [12].

The results from our study seem counterintuitive, considering data from multiple laboratory studies suggesting the shorter survival of red blood cells with a prolonged storage period. Storage lesion-associated increased hemolysis is probably not severe enough to cause a significant difference in the post-transfusion hemoglobin that can be detected by standard laboratory techniques. It is noteworthy that we excluded patients with evidence of hemolytic anemia to limit its role as a confounding factor. The immune system of patients with hemolytic anemia is already primed to mount an immune response to the red blood cell. Transfusing old blood that is immunologically susceptible can lead to exaggerated hemolysis in such patients.

Our study has limitations. This was a retrospective study with a limited patient population. Both groups were similar in patient characteristics but there is still a possibility of confounding factors that we may not have taken into account. Although we excluded patients who received intravenous fluid bolus, we did not account for the maintenance intravenous fluids that can potentially effect hemoglobin level. We cannot extrapolate our findings to all patients needing blood transfusions, as we excluded patients who received multiple transfusions and patients with evidence of hemolysis or bleeding. We only looked at hemoglobin levels within 24 hours of transfusion and cannot exclude the possibility that there could be a difference in the hemoglobin level at an extended follow-up period. This is, however, unlikely since most of the post-transfusion hemolysis occurs within 24 hours. We propose that a prospective clinical trial in controlled settings is required to further validate our findings. In future trials, we recommend following patients' hemoglobin for an extended period of time and comparing the PRBC requirement in patients who received new blood versus old blood.

## Conclusions

In conclusion, although most laboratory studies have shown the storage lesion to be associated with shorter survival and increased hemolysis after transfusion, we found that it does not translate into a significant difference in the hemoglobin level after transfusing new blood versus old blood. We do not recommend preferentially transfusing blood with a short storage period based on clinical condition or need for multiple transfusions.
